# Ultrasound Features in Gout: An Overview

**DOI:** 10.3390/medsci12030037

**Published:** 2024-07-31

**Authors:** Cristina Dorina Pârvănescu, Andreea Lili Bărbulescu, Cristina Elena Biță, Ștefan Cristian Dinescu, Beatrice Andreea Trașcǎ, Sineta Cristina Firulescu, Florentin Ananu Vreju

**Affiliations:** 1 Department of Rheumatology, Faculty of Medicine, University of Medicine and Pharmacy of Craiova, 200349 Craiova, Romania; parvanescu.reumatologie@gmail.com (C.D.P.); cristina.gofita@umfcv.ro (C.E.B.); beatrice_med@yahoo.com (B.A.T.); sineta.firulescu@gmail.com (S.C.F.); 2 Department of Pharmacology, Faculty of Medicine, University of Medicine and Pharmacy of Craiova, 200349 Craiova, Romania; florentin.vreju@umfcv.ro

**Keywords:** gout, ultrasound, double contour, tophi, snowstorm sign

## Abstract

The accurate diagnosis of gout frequently constitutes a challenge in clinical practice, as it bears a close resemblance to other rheumatologic conditions. An undelayed diagnosis and an early therapeutic intervention using uric acid lowering therapy (ULT) is of the utmost importance for preventing bone destruction, the main point of managing gout patients. Advanced and less invasive imaging techniques are employed to diagnose the pathology and ultrasonography (US) stands out as a non-invasive, widely accessible and easily reproducible method with high patient acceptability, enabling the evaluation of the full clinical spectrum in gout. The 2023 EULAR recommendations for imaging in diagnosis and management of crystal-induced arthropathies in clinical practice state that US is a fundamental imagistic modality. The guidelines underline its effectiveness in detecting crystal deposition, particularly for identifying tophi and the double contour sign (DCS). Its utility also arises in the early stages, consequent to synovitis detection. US measures of monosodium urate (MSU) deposits are valuable indicators, sensitive to change consequent to even short-term administration of ULT treatment, and can be feasibly used both in current daily practice and clinical trials. This paper aimed to provide an overview of the main US features observed in gout patients with reference to standardized imaging guidelines, as well as the clinical applicability both for diagnosis accuracy and treatment follow-up. Our research focused on summarizing the current knowledge on the topic, highlighting key data that emphasize gout as one of the few rheumatological conditions where US is recognized as a fundamental diagnostic and monitoring tool, as reflected in the most recent classification criteria.

## 1. Introduction

Gout is the most common crystal-induced arthritis, with documented increasing incidence and prevalence during the last decades. Among the most significant risk factors are obesity, dietary habits, hypertension, altered renal function, or diuretic treatment. It is a disease characterized by abnormal purine metabolism and urate excretion, with consequent deposition of MSU intra or peri-articular and future local destructive evolution along with multisystem involvement. Crystal deposition leads to episodic gout flares followed by chronic tissue inflammation and local irreversible changes [1].

The certain diagnosis of gout frequently constitutes a challenge in clinical practice, as it shows a close resemblance to other rheumatologic conditions, such as osteoarthritis, or inflammatory autoimmune pathologies, such as rheumatoid arthritis. Moreover, the appearance of tophus at clinical examination can present similar characteristics to tumors or calcium pyrophosphate dihydrate deposition (CPPD) [2]. An undelayed diagnosis and early therapeutic intervention through uric acid lowering therapy (ULT) is of the utmost importance for preventing bone destruction, the main point of managing gout patients [3].

Although detecting MSU crystals in synovial fluid remains the diagnostic gold standard, advanced and less invasive imaging techniques are employed for diagnostic purposes and constitute basic tools for clinical practice [4]. Conventional radiology has been traditionally used for decades, as a first imaging modality, economical and accessible, with the major disadvantage of a limited overview in acute stages. It can detect certain changes, such as bone erosions, narrowing articular spaces, or the presence of tophus [5]. Dual-energy computed tomography (DECT) was first reported in 2007 and employs X-ray beams at different energies, with four primary methods represented by sequential scanning, dual source, rapid kilovoltage switching, and dual layer. The differences in photoelectric absorption determine the distinction between urate and bone on the achieved images and allow subclinical tophus deposits to be identified. Its appliance in clinical practice exerts various specificity and sensitivity rates in the published researches, validated by 2018 EULAR recommendations [4,5]. Multi-energy spectral photon-counting computed tomography (SPCCT) has been recently approached as a novel imaging method in crystal-induced arthropathies, as it can distinguish between MSU, calcium pyrophosphate, and hydroxyapatite crystal deposits ex vivo [6]. Magnetic resonance imaging (MRI) finds its use in clinical practice in cases of unusual settings of gout. The images can picture the inflammatory aspect of gouty arthropathy, including synovitis, tenosynovitis, and edematous soft tissue inflammation [7]. Musculoskeletal ultrasonography (US) stands out to be a non-invasive, widely accessible, and easily reproducible method with a high patient acceptability, that can be consequently approached in daily clinical practice. Using a high-frequency transducer by an experienced examiner, the method makes it possible to detect crystal deposition and distinguish between other possible pathologies [8]. The recent 2023 EULAR recommendations on imaging in diagnosis and management of crystal-induced arthropathies in clinical practice state that imaging can offer details regarding crystal deposition, inflammation, and structural damage, features that are not always associated with clinical manifestations. For both diagnostic and monitoring purposes, US is a fundamental recommended imagistic modality, with evidence demonstrated for crystal deposition, especially tophi and DCS. Its utility also arises in the early stages, consequent to synovitis detection. Revealing specific signs of MSU crystal deposition by US offers a high diagnostic capacity and does not require synovial fluid analysis [9].

This paper aims to provide an overview of the main US features observed in gout patients with reference to standardized imaging guidelines, as well as the clinical applicability both for diagnosis accuracy and treatment follow-up. Our research focused on summarizing the current knowledge on the topic, highlighting key data that emphasize gout as one of the few rheumatological conditions where US is recognized as a fundamental diagnostic and monitoring tool, as reflected in the most recent classification criteria.

## 2. US Findings in Gout

US achieves images on structure description by delivering soundwaves and visualizing bone erosions and soft tissue details throughout acoustic reflections. It constitutes a valuable tool for diagnosing gout, as it can detect early MSU crystal deposits in joint structures like hyaline cartilage surfaces and the synovium. It is also useful for evaluating synovial thickness, synovial effusion, and bone erosion. Additionally, power Doppler US can assess synovial inflammation [10]. In gout, MSU crystal deposits reflect ultrasound beams more strongly than surrounding tissues, such as unmineralized hyaline cartilage or synovial tissue. As a result, crystalline material can be detected by US as a bright, hyperechoic signal [11].

US findings in gout patients commonly include synovitis, tenosynovitis, and subcutaneous edema during joint attacks, along with crystal deposits in joints and tendons. To standardize descriptions of these lesions, the Outcome Measures in Rheumatology (OMERACT) Ultrasound Working Group developed consensus definitions. The validation process established US definitions for the four main structural lesions in gout: tophi (larger collection of crystals), aggregates (urate burden), double contour sign, and erosions (structural damage) [11] (Table 1).

**Figure 1:**
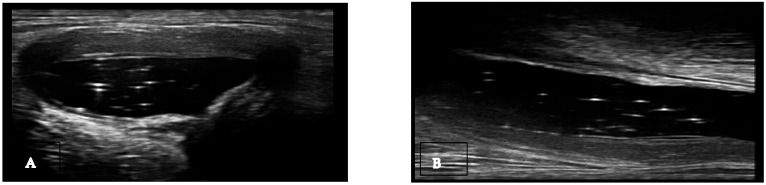
Baker cyst in transverse (**A**) and longitudinal (**B**) view with the presence of multiple hyperechoic spots “snowstorm sign”.

Non-specific signs. Gout is an inflammatory disease that manifests through episodes of acute arthritis. During the progression of the disease, urate arthropathy may occur, accompanied by joint damage. Similar to rheumatoid arthritis and other erosive rheumatism, joint effusion, synovial hypertrophy, and non-specific bone erosions can be found. Joint effusion can be found in gout (Table 1), and the presence of hyperechoic spots (snowstorm sign) in the synovial fluid suggests a crystalline pathology but is not specific to gout (Figure 1) [13]. Synovitis, including Doppler activity, is not considered an elementary lesion for gout because it is not specific enough to define the condition. Synovial hypertrophy and hypervascularization are not specific to gout, but their association with hyperechoic spots (bright focal areas) in the synovium strongly suggests gout. When assessing synovitis and tenosynovitis in gout patients, the definitions validated by OMERACT ultrasound group for rheumatoid arthritis are used. Synovial hypertrophy is another non-specific sign that can be found in gout (Table 1). Tenosynovitis is defined as hypoechoic or anechoic thickened tissue within the tendon sheath, with or without fluid, seen in two perpendicular planes and potentially exhibiting a Doppler signal [11,12,13].

Specific signs. Several US characteristics have been described in gout, and some appear to be highly specific [14].

**Figure 2:**
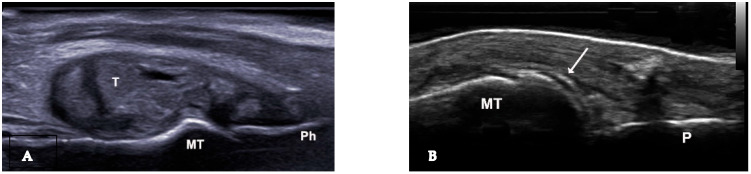
(**A**). Urate deposits, grade 3 (definite and severe) visible in longitudinal view at the first metatarsophalangeal joint. (**B**). Urate deposits at de surface of the metatarsal bone cartilage—double contour sign. MT—metatarsal bone, P—phalanx, T—intraarticular tophus, arrow—double contour sign.

**Figure 3:**
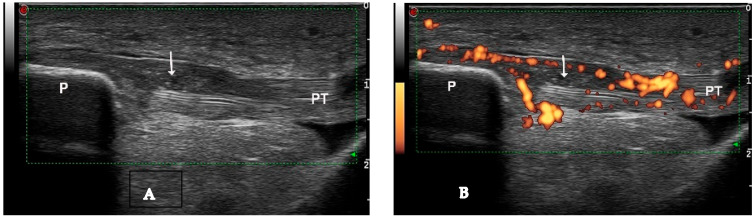
Intratendinous soft tophus visible in longitudinal view of the proximal insertion of the patellar tendon ((**A**)—grayscale, (**B**)—Power Doppler mode). P—patella, PT—patellar tendon, arrow—intratendinous soft tophus.

**Figure 4:**
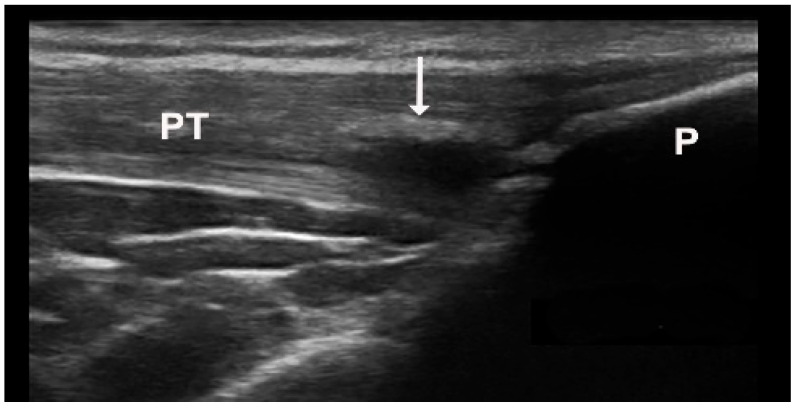
Intratendinous hard tophus visible in longitudinal view of the distal insertion of the patellar tendon. Notice the posterior shadowing from the tophus. P—patella, PT—patellar tendon, arrow—intratendinous hard tophus.

**Figure 5:**
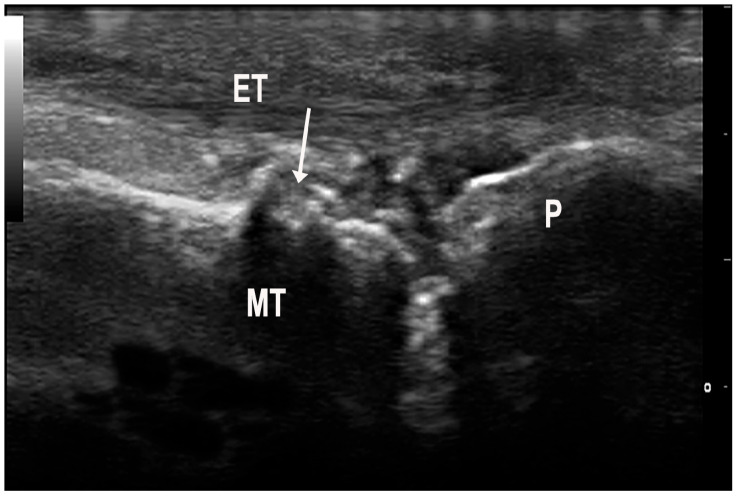
Erosions (arrow) of the metatarsal head (MT) in a patient with gout. To note a grade 2 urate aggregate at the level of the metatarsophalangeal joint. ET—extensor tendon, P—phalanx.

Tophi and aggregates, which can be observed in joints and soft tissues such as tendons and bursae, represent larger collections of MSU crystals and may cause bone erosions by invading bone. US detects a tophus as a heterogeneous mass with various US appearances (Figure 2A, Figure 3A,B and Figure 4), with the possibility of hyper-echoic appearance due to the presence of hyperechoic spots (Table 1). The association with posterior attenuation of the ultrasound or total acoustic shadowing depends on the density of the tophus. Thus, a cloudy mass in a joint, with hyperechoic spots and a small anechoic rim, strongly suggests gout. Moreover, US is used to visualize tophi in symptomatic joints, including the first metatarsophalangeal (MTP) joints (dorsal and lateral planes), knee (quadriceps, patellar, and lateral ligaments), and ankle tendons (Achilles tendon, anterior tibial tendon). In small joints, intra-articular tophi are often associated with bone erosions [15]. Sometimes, tophi can be poorly defined and extend across multiple fascial planes. Tophi that appear hypoechoic on imaging, without posterior shadowing, are described as “soft tophi”, while long-standing tophi that obstruct the visualization of underlying structures are known as “hard tophi” [16]. In 2018, an international expert consensus was reached using OMERACT methodology for a re-definition of aggregates and for the development of a semi-quantitative US scoring system for gout lesions associated with MSU depositions (Table 2) [17]. Bone erosions in gout are defined similarly to those in rheumatoid arthritis. However, in gout, bone erosions are often found extra-articularly, and their distribution, rather than the appearance of a single erosion, is a characteristic of the disease [13] (Figure 5).

**Figure 6:**
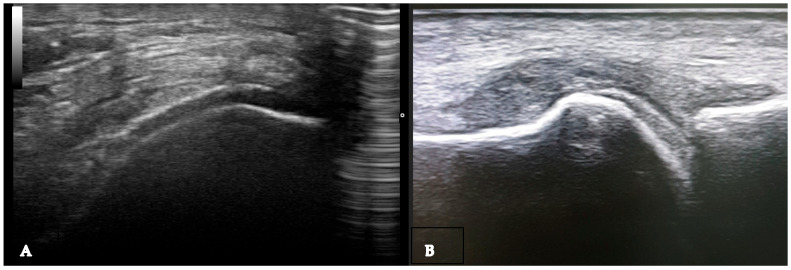
Ultrasound features of “double contour sign” visible on the cartilage surface of the femur (**A**) and first metatarsal bone (**B**).

The double contour sign (DCS) indicates deposits of MSU crystals on cartilage surfaces, distinguishable from calcium pyrophosphate crystal deposits typically found within cartilage, highly specific for gout (Figure 2B and Figure 6A,B). Due to MSU crystal deposition, the reflectivity of the chondrosynovial interface is no longer angle-dependent, allowing for easy panoramic visualization of the entire chondrosynovial interface [18,19]. Like the tophus, the DCS should be sought in symptomatic joints, including the first metatarsophalangeal joints (dorsal and palmar planes) and the trochlear cartilage of the knees (suprapatellar plane in maximum flexion). The DCS may be less visible in thin cartilage (tarsal joints) or damaged cartilage, such as in osteoarthritis. Additionally, certain ultrasound features can be mistaken for a DCS, leading to false positives due to several factors: first, the normal hyperechoic appearance of the synovium, where the hyperechoic band appears regular, like a line drawn with a pen. A “true” DCS adheres to the cartilage during dynamic movements. Second, the presence of joint effusion enhances the echo of the posterior wall (increased ultrasound propagation) and may accentuate the normal hyperechoic appearance of the synovium. Finally, thin cartilage (small joints and/or associated osteoarthritis) with chondrocalcinosis and calcium deposits often localized in the intermediate layer of the cartilage [14,16]. Regarding the diagnostic performance of the DCS sign, a recent study by Cipolletta et al. emphasized that dynamic examination significantly improves the effectiveness of US in differentiating between gout and calcium pyrophosphate dihydrate CPPD crystal arthritis. Their results indicated that, with dynamic examination, the DCS sign moved with the cartilage in all cases of gout, whereas it moved in the opposite direction in CPPD patients. [20]. In 2022, Filippou et al. conducted an anatomical cadaver study to compare US DCS findings with pathological features, aiming to evaluate how crystal location affects ultrasound characteristics. The study was performed on upper limb joints until calcium pyrophosphate (CCP) deposits were detected according to OMERACT criteria. The affected joints were then examined pathologically, with crystal deposition described. The findings concluded that monosodium urate (MSU) crystals in gout are located directly on the chondral surface and cause the DCS sign to move with the cartilage. In contrast, CCP crystals are found in capsules and/or ligaments, above the hyaline cartilage, and do not exhibit dynamic sliding during US examination [21].

In gout patients, the most common location for all four gout lesions is represented the MTP1 joints, with a DCS mean count of 0.8 and more than one tophus, aggregate, and erosion on average. MTP2–4 and talocrural joints typically showed DCS and aggregates. MTP5 joints frequently had erosions. The knees were the second most common site for DCS. In MCP joints, aggregates were the most common finding, while DCS was less common, and tophi or erosions were rare. Wrists commonly had aggregates. Tendon involvement was relatively common, with tophi most often found in the peroneus tendons, followed by the proximal patella, triceps, distal patella, quadriceps, and Achilles tendon insertions. Extensor tendons of the wrist and tibialis posterior tendon involvement were very rare [17].

The presence of tophi, DCS, and the snowstorm sign are directly linked to serum urate acid (SUA) levels. Research has shown that the DCS disappears when SUA levels are kept below 6 mg/dl for over six months, although tophi take longer to dissolve [22]. These US signs appear in a specific sequence, with tophi typically developing late, after a median disease duration of 12.5 years. This may explain the slightly lower sensitivity and similar specificity in joint-based evaluations compared to person-based evaluations [23]. For established gout patients, the snowstorm sign appears earlier, with a median disease duration of 2 years, while those without this sign had a median duration of 5.5 years, indicating its lower diagnostic value in long-term gout. The DCS appears between the appearance of the snowstorm sign and tophi. Thus, different patient populations with varying disease durations contribute to the heterogeneous results among studies [24].

What sites should be scanned in gout?

Although there are well-known and validated sites to be examined in crystal-induced arthropathies (MTP1 in gout, knees and wrist in CCPD, or shoulder in basic calcium phosphate deposition-BCPD), several locations have been shown to exhibit US signs of MSU deposition [9]. It is also important to note the importance of different disease stages in the appearance of certain US findings. A recent paper published by Cipolletta et al. aimed to assess the most effective US methodology for diagnosing gout or CPPD in patients with acute arthritis. The study, which analyzed 32 gout and 30 CCPD patients, compared to 99 controls, found that examining knees and first MTP joint in gout, and both knees and wrist in CPPD, along with the symptomatic joint, provided high and feasible sensitivity and specificity. This approach proved more effective than focusing only on the symptomatic joint, identifying key joints to scan in cases of recent onset monoarthritis [25].

Extra-articular gout commonly involves tendons, with monosodium urate (MSU) crystal deposits frequently found in the Achilles, patellar, peroneal, and flexor or extensor tendons of the hand. Detecting tendon involvement is crucial due to the risk of spontaneous rupture, which can significantly impact a patient’s quality of life. A multicenter descriptive study involving 80 gout patients aimed to evaluate the prevalence of Achilles, quadriceps, and patellar tendon involvement compared to individuals with osteoarthritis or asymptomatic marathon runners. The results showed a significant presence of intra-tendinous aggregates and tophi, with the highest percentage located in the Achilles tendon [26]. Another relevant research aimed to analyze the US features of Achilles tendon in patients with tophaceous gout compared to healthy individuals. The study found a high prevalence of tophi, intra-tendinous PD signal, and hyperechoic spots, with an extremely low incidence of structural damage. Additionally, only minimal erosions were described at the calcaneal entheseal site [27].

MSU crystal deposition can also involve the axial skeleton, leading to spinal compression and subsequent clinical symptoms. A cross-sectional study of gout patients, compared to healthy subjects, aimed to assess the presence and volume of MSU deposits in the lumbosacral area using DECT imaging, with both default and specifically adjusted to MSU settings. The results demonstrated a substantial amount of MSU deposits in the lumbosacral region, despite the use of precise settings designed to eliminate interference from other signals. These findings emphasize the need to consider MSU deposition as a potential diagnosis in patients with gout who present with axial symptoms, such as pain or neuropathic features, indicating that gout can affect not only peripheral joints but also axial sites [28].

US scoring systems. In addition to developing and validating consensus-based definitions of gout characteristic US lesions, to fulfill the requirements of an OMERACT Imaging Measurement Instrument, the method requires a stepwise selection and development of a well-defined, standardized scoring system for grading lesion severity at site level. In 2018, an international expert consensus was reached using OMERACT methodology to develop a semi-quantitative US scoring system for gout lesions associated with MSU depositions. The scoring system for DCS, tophus, and aggregates was divided into four categories and established as: 0—absent; 1—possible; 2—definite but minimal; 3—definite and severe [17].

US in patients with asymptomatic hyperuricemia. A significant number of studies reported the presence of several US changes subsequent to crystal deposition in patients with asymptomatic hyperuricemia (AH). Although no systematic US scanning is recommended in AH, periodic evaluation enables the detection of patients at risk not only for gout flares but also for cardiovascular events consequent to crystal-induced chronic inflammation [29]. In 2011, Pineda et al. performed a cross-sectional study involving 40 patients with AH and found the DCS at the MTP joint in 25% of the cases. Their results also included additional description of tophi or entesophytes at sites such as Achilles or patellar tendon sites was also mentioned by their results [30]. In 2021, Cao et al. reported the presence of MSU crystals in 25.58% of the 43 patients with AH included in their analysis [31]. However, a 2024 study published by Shao Q et al. found a lower percentage, detecting MSU crystal deposition in 17 of 81 AH patients, mostly in the MTP l joint, ankle, and peroneus-longus and brevis tendons [32]. Another relevant study, published in 2024, assessed the impact of various US protocols on detecting MSU deposition in a group of 77 AH patients. The results found a median of one location with tophi and one with aggregates; the percentage of crystal deposition varied between 23.4% and 87%, with PD signal present in 19.5% of the cases, and erosions observed in 28.4% of the patients [29]. Additionally, a recent study evaluated the association between hyperuricemia and US-detected hand synovitis in a large cohort of over 3200 randomly selected subjects. The study reported a higher prevalence of hand synovitis, demonstrated in grayscale, for patients with AH when compared to those with normal UA levels, and identified a direct association between hyperuricemia and this US finding [33].

## 3. US Diagnostic Accuracy

An early, accurate diagnosis and individualized therapeutic approach prevents the inherent destructive articular process consequent to MSU deposition, with a major impact on associated morbidity and mortality rates, as well as on patients’ quality of life. Advanced imaging techniques enable clinicians to have a proper overview of the pathological process, providing a new tool to explore the disease and appreciate its future evolution [34,35]. US has attracted more and more attention during the past decades, consequent to its outstanding development, extensive availability, and easy reproducibility, making it an indispensable tool for our current practice. An important number of studies have focused on describing the diagnostic accuracy of US in gout, with important results that require attention and applicability for gout patients [25,26,27,28,29,30,31,32,33,34,35].

The meta-analysis conducted by Lee et al., published in 2018, included 11 studies, 938 patients with gout and 788 controls, and revealed important results regarding the overall sensitivity and specificity of US. Their analysis demonstrated that US was more specific (89%) than sensitive (65.1%). Taken together, the diagnostic performance was good, with an AUC of 0.858. Moreover, when analysis was divided in subgroups, depending on diagnostic criteria, number of patients and study design, the diagnostic performance was maintained. An important conclusion of the research was that a high specificity was achieved by revealing certain US characteristics such as tophus, snowstorm, and erosions, although they displayed a limited sensitivity [36]. In contrast, the DCS proved to be a highly sensitive and specific finding, the only one included in the 2015 gout classification criteria developed by the ACR and EULAR [37]. An important mention regarding this US characteristics of gout is that is limited regarding small articular sites (PIP, DIP), mainly due to poor acoustic window. For these locations, an accurate diagnosis is established in the presence of tophus deposits. Overall, the appearance of typical US characteristics provides a good sensitivity and a high specificity for diagnostic purposes [36].

The research of Zhang et al., published in 2018, included 13 studies that confirmed that highly specific features of gout, DCS, tophi and snowstorm sign, closely related to the deposit of MSU crystals, were consistent with their results, with a high specificity, over 0.90 for all, regarding establishing a certain diagnosis of gout. Additionally, lower sensitivities, of 0.66 for DCS, 0.56 for tophi and 0.31 for snowstorm sign, constitute an argument for the mention that the lack of one of the characteristics does not exclude the diagnostic probability of gout. Simultaneously interpretation of these US features improved the diagnostic accuracy compared to the evaluation based on each joint, for detecting the presence of DCS and tophi, as specific US signs in gout [38]. Previous studies have confirmed the first MTP joint and knee constitute the most frequently involved articular sites in gout [39,40,41,42,43,44]. The aforementioned meta-analysis reported that the sensitivity in articular site-based evaluation is slightly higher but not statistically different compared to an overall evaluation of each patient. Moreover, each site’s evaluation specificity is significantly lower compared to the overall one [38]. One possible explanation for this result might be represented by the fact that the patients with chronic or subacute stages of gout were likely to have false negative findings on preestablished articular sites when performing the US examination. Additionally, the included studies had a significant number of patients with CPPD that negatively influenced the observed specificity. The false negative cases may appear for several reasons. The level of SUA directly impacts the presence of tophi, DCS, and snowstorm sign [45,46]. In this direction, Das et al. reported that a therapeutic success, with the achievement of SUA below 6 mg/dl for at least 6 months, determines the disappearance of DCS, but not for tophi [18]. Additionally, several scientific reports proved that the appearance of specific signs has a particular, sequential order. The longer duration is found for tophi, which can be observed in patients with a mean duration of the disease of 12.5 years [23,45], fact that constitutes a cause for the lower sensitivity, but similar specificity associated with each articular site US evaluation when compared to overall sites US evaluation. A different report was published by Elsaman et al., that concluded that in cases of established, chronic gout, the appearance of snowstorm sign was associated with a mean disease duration of 2 years and 5 years for those in which US evaluation did not reveal this feature, a conclusion that underlines the fact that this early characteristic has a lower diagnostic value in patients with a long duration of the disease [24]. Additionally, an important observation was that in cases with DCS on US, the mean duration was in between the one calculated for the presence of snowstorm and tophi [24]. The different results reported by the studies included in the meta-analyses are a consequence of the heterogeneity of the populations and different disease durations.

The meta-analysis published in 2015 by Ogdie et al., clarified the pooled diagnostic accuracy. Their research included eleven studies, all set in secondary care, with a mean gout disease duration of at least 7 years. Their research lacks comprising all the US features, part of them providing data about DCS, others regarding tophi, and at utmost importance, the final analysis did not differentiate between overall articular evaluation and articular-site based ones. Additionally, the overall US examination includes multiple joints, while articular-site based ones only include symptomatic ones [23].

A recent meta-analysis, published in 2022 by Shang et al., aimed to compare the diagnostic accuracy of DECT over US for gout, by analyzing data from both imagistic methods separately. A total of 14 of the included studies reported data regarding US examination, 10 only reported on DECT, and four were based on a comparison between the DECT and US examination [47]. According to DCS sign, eleven studies provided data and revealed a sensitivity and specificity of 0.7 and 0.95, respectively [5,23,24,43,48,49,50,51,52,53,54]. For the tophus identification, eight studies [23,24,43,48,49,50,52,53] provided data on the diagnostic accuracy of US and indicated that the pooled sensitivity and specificity were 0.57 and 0.99. Regarding overall findings, 11 studies [9,23,24,49,52,55,56,57,58,59,60] provided data and showed a specificity and sensitivity of 0.84. A subgroup analysis of patients with a disease duration of a maximum 2 years, considering the overall US characteristics, showed a sensitivity and specificity of 0.93 and 0.8.

In 2020 Zhang et al. compared the diagnostic value of US and DECT in detecting MSU deposits in patients with different stages of acute gouty arthritis. Their results evidenced that US and DECT had similar sensitivities in middle and late-stage groups but in early stages, US showed a significantly higher sensitivity (66.7%) compared to DECT (26.6%). The pooled outcomes indicated that the disease course strongly affected the diagnostic accuracy of both modalities [61]. US allows evaluation of the full clinical spectrum in gouty arthritis from its earliest to its most advanced characteristics [5]. Thus, the sensitivity and AUC of DECT were decreased in patients with short disease course because small MSU deposits might remained undetected in the early stages of gout [62]. In a similar manner, Bongartz et al. [62] and Jia et al. [63] showed that the sensitivity of DECT was lower in recent onset or gout flare, as Lamers-Karnebeek et al. reported a sensitivity of 96% for US in acute gout [52].

The call for more research is mandatory in order to completely compare the diagnostic performance of US versus DECT for patients at first gout flare or during early phases when tophaceous deposits are absent. As the main scope of diagnosis is to identify and properly manage the pathology before the appearance of erosions, the necessity of establishing the most accurate method is highly necessary. However, there must be taken under consideration the accessibility, reproducibility, and level of comfort for the patients, along with the level of radiation exerted by DECT examination.

## 4. US Evaluation for Urate-Lowering Therapy (ULT) Follow-Up

Conventional monitoring of therapy efficacy in gout has relied on clinical assessment along with periodical evaluation of SUA levels. As US, as a valuable imaging method, improved over time and showed outstanding utilities in clinical practice, along with an extended availability and reproducibility, it is currently used for periodic assessment. OMERACT described important endpoints in gout therapeutic management, represented by the MSU load and the decrease in urate deposits, both visualized by US evaluation [64].

A study performed by Ebstein et al., on a group of 79 patients, with US evaluations at baseline, 3 months and 6 months, during ULT, reported that DCS and tophus significantly decreased during treatment. In particular, DCS represented an early sign of therapeutic success, with a significant change after the first 3 months of treatment. Tophi were observed to exert reductions after 6 months of therapy administration. Their analysis also showed a significant decrease in US features of gout among patients with the lowest SUA level [65]. Peiteado et al. reported similar results in 2019, with a significant parallel improvement in the SUA levels and US features found at the follow-up assessments [66]. Das et al. performed an observational study on 38 patients, intending to monitor US signs of MSU crystals deposition after initiation of ULT and concluded that DCS and hyperechoic spots disappeared after 6 months and 5.7 months, respectively and SUA normalization was the only significant predictor of DCS disappearance [22].

In 2023, Yuan et al. published a report regarding the effectiveness of uric acid-lowering therapy in 215 patients with gout over one year using US as a monitoring method. Their analysis was divided into two directions: treat-to-target (TTG) and treat-to-non-target (TNTG) subgroups. Data showed that after one year of ULT the area, long diameter, and short diameter of tophus and SQUS-DCS in the TTG subgroup reduced significantly (DCS faster than tophus). Although DCS decreased in TNTG, DCS decreased faster in TTG than in TNTG [67].

El-Mallah et al., in 2022, aimed to evaluate the changes in ultrasound of 43 gout patients’ knee and 1st metatarsophalangeal joint (MTP1) after initiation of urate-lowering therapy (ULT) drugs in the six-month period and concluded that patients that reached the target SUA level showed higher disappearance of DC sign and a decrease in tophus size. The percentage of tophus size in 6 months was 26.4% and 3% for DC sign disappearance, which was more at MTP1 [68].

In 2020, Hammer et al. published one of the most extended US studies regarding imaging changes consequent to TTG approach. A total of 209 patients were evaluated at baseline, 3, 6, and 12 months, and a semiquantitative scoring system of basic features (DCS, tophi and aggregates) was used to reckon the evolution during follow-up. Their results showed that DCS was the first variable that improved and exerted the highest sensitivity. It is important to note that at their 12 months evaluation for almost 50% of the patients, DCS was not present on US imaging. This observation can be explained by the fact that MSU crystals are deposited near cartilage and in close contact with synovial fluid, a location where the UA levels decrease fast, consequent to ULT. It is important to note that MTPI was the most common site for MSU deposit, and the erosions pointed in this location were significantly related to the other US findings [69].

Another relevant research, published by Christiansen et al., aimed to assess the sensitivity of structural gout lesions changes as defined by OMERACT US group in a cohort of 50 patients undergoing ULT. Their results disclosed a relevant decrease in DCS and tophus scores during treatment, whereas the aggregate sum score diminished significantly only from 3 to 6 months; the erosion sum score did not exert any notable change during treatment. Another important observation was that all four features were most commonly revealed in MTPI joints, while DCS alone was most frequently observed in the knee. These two sites represented the locations the worthiest to note regareding score changes [70].

Contemporary published data underline that US constitutes an effective tool for monitoring dynamic changes in tophus and DCS. US measures of MSU deposits are valuable indicators, sensitive to change consequent to even short-term administration of ULT treatment, and can be feasibly used both in current daily practice and clinical trials. Although a complete examination of multiple articular sites may be time-consuming, its current use exerts an additional motivational appliance both for patients and physicians and helps to augment treatment adherence, an important point of each individualized therapeutic approach.

## 5. Conclusions

US has become one of the main imaging tools used in the management of gout patients during the past decades. This is due to its availability and developments in the standardized approach which increases its reproducibility. US constitutes an essential imaging test for current practice, which enables a high diagnostic accuracy and can be easily integrated in the therapeutic follow-up.

## Figures and Tables

**Table 1:** OMERACT definitions for US findings in gout [11,12].

Specific Findings	OMERACT Definitions
Double contour sign	“Abnormal hyperechoic band over the superficial margin of the articular hyaline cartilage, independent of the angle of insonation and which may be either irregular or regular, continuous or intermittent and can be distinguished from the cartilage interface sign”.
Aggregates	“Heterogeneous hyperechoic foci that maintain their high degree of reflectivity even when the gain setting is minimized or the insonation angle is changed and which occasionally may generate posterior acoustic shadow”.
Tophus	“A circumscribed, inhomogeneous, hyperechoic and/or hypoechoic aggregation (which may or may not generate posterior acoustic shadow) which may be surrounded by a small anechoic rim”.
Bone surface changes (erosions)	“An intra- and/or extra-articular discontinuity of the bone surface (visible in 2 perpendicular planes)
**Non-specific findings**	
Synovial fluid	“Abnormal hyperechoic or anechoic (relative to subdermal fat, but sometimes may be isoechoic or hyperechoic) intra-articular material that is displaceable and compressible; does not exhibit Doppler signal”.
Synovial hypertrophy	“Abnormal hypoechoic (relative to subdermal fat, but sometimes may be isoechoic or hyperechoic) intra-articular tissue that is not displaceable and poorly compressible; may exhibit Doppler signal”.
Power Doppler signal	

**Table 2:** New definition of aggregates according to OMERACT [17].

Overarching principle	“*The aggregates can only be scored in a patient if other ultrasound features suggestive of gout such as DC and/or tophus are present/have previously been present at patient level and if the aggregates are not located inside a tophus*”.
Aggregates: [independent of location (intraarticular/intratendinous)]	“*Bright hyperechoic, isolated spots too small to fulfil the tophus definition and characterized by maintaining their high degree of reflectivity when the insonation angle is changed*”

## Data Availability

Not applicable.
